# Single-cell magnetotaxis in mucus-mimicking polymeric solutions

**DOI:** 10.3389/fmicb.2024.1436773

**Published:** 2024-07-18

**Authors:** Brianna Bradley, Carlos Escobedo

**Affiliations:** Department of Chemical Engineering, Queen’s University, Kingston, ON, Canada

**Keywords:** magnetotactic bacteria, microfluidics, magnetotaxis, viscous fluid, non-Newtonian fluid, polymeric solutions, mucus

## Abstract

Magnetotactic bacteria (MTB) are promising candidates for use as biomicrorobots in biomedical applications due to their motility, self-propulsion, and the ability to direct their navigation with an applied magnetic field. When in the body, the MTB may encounter non-Newtonian fluids such as blood plasma or mucus. However, their motility and the effectiveness of directed navigation in non-Newtonian fluids has yet to be studied on a single-cell level. In this work, we investigate motility of *Magnetospirillum magneticum* AMB-1 in three concentrations of polyacrylamide (PAM) solution, a mucus-mimicking fluid. The swimming speeds increase from 44.0 ± 13.6 μm/s in 0 mg/mL of PAM to 52.73 ± 15.6 μm/s in 1 mg/mL then decreases to 24.51 ± 11.7 μm/s in 2 mg/mL and 21.23 ± 10.5 μm/s in 3 mg/mL. This trend of a speed increase in low polymer concentrations followed by a decrease in speed as the concentration increases past a threshold concentration is consistent with other studies of motile, flagellated bacteria. Past this threshold concentration of PAM, there is a higher percentage of cells with an overall trajectory angle deviating from the angle of the magnetic field lines. There is also less linearity in the trajectories and an increase in reversals of swimming direction. Altogether, we show that MTB can be directed in polymer concentrations mimicking biological mucus, demonstrating the influence of the medium viscosity on the linearity of their trajectories which alters the effective path that could be predefined in Newtonian fluids when transport is achieved by magnetotaxis.

## 1 Introduction

Microrobots have great potential to transform medical practices, enabling less traumatic therapeutic and diagnostic procedures and the potential development of previously impossible medical procedures. However, powering and engineering the locomotion of artificial microrobots is difficult, since they must be able to store, harvest, and transduce the power into motion on a small-scale, which places strict limitations on their design (Nelson et al., 2010). To overcome this challenge, motile microorganisms may be used, taking advantage of their self-propulsion capabilities. Specifically, magnetotactic bacteria (MTB) could be used as biological microrobots, but it is essential to establish reliable methods for controlling their navigation to use them in biomedical applications.

MTB are a group of bacteria with an internal chain of magnetosomes, an organelle consisting of nanometer-sized magnetic crystals (Bazylinski and Frankel, 2004). The chain of magnetosomes causes the bacterium to passively align and actively swim with magnetic field lines, a navigation phenomenon called magnetotaxis (Frankel et al., 2006). This behavior allows for the directed navigation of the cells using an applied magnetic field, making them a promising candidate for use in targeted therapy. MTB have been shown to be capable of penetrating into a multicellular tumor spheroid (Mokrani et al., 2010) and delivering drug-loaded nanoliposomes into hypoxic regions of tumors (Felfoul et al., 2016). Additionally, MTB have been used as hyperthermia agents to treat cancer tumors, in which an alternating magnetic field heats the magnetic nanoparticles and this increase in temperature results in cell death of the surrounding cancerous cells (Gandia et al., 2019).

When MTB are used in the body for targeted therapies, they may encounter non-Newtonian fluids, depending on the target location, such as blood plasma (Brust et al., 2013; Varchanis et al., 2018) or various types of mucus, such as that of the gastrointestinal, respiratory, and urogenital tract, as well as the peritoneal surface of intra-abdominal organs (Lai et al., 2009). A non-Newtonian fluid has variable viscosity dependent on shear, whereas a Newtonian fluid has a constant viscosity. An understanding of the capability of directing MTB using a magnetic field through non-Newtonian fluids will be essential to their use for targeted therapies.

Here, we investigate the effects of different concentrations of a non-Newtonian polymer fluid on the magnetotactic abilities of MTB, mimicking the microenvironments they would encounter when swimming in bodily fluids, in order to understand specific aspects of their motion at single-cell level. A microfluidic device was designed, fabricated and used in the experiments to control the flow and restrict the region of observation of bacterial motility. Swimming characteristics of the MTB in different concentrations of polymer solution under a constant, linear magnetic field produced by a custom electromagnetic-microfluidic platform were analyzed including speed, trajectory angle, linearity, and run-reverse motion.

## 2 Materials and methods

### 2.1 Viscosity characterization

The viscosity with respect to shear rate for concentrations from 1 to 6 mg/mL of polyacrylamide (PAM, FLOPAM FA 920 VHM, SNF Canada) dissolved in the MTB growth medium was measured using a DV-E viscometer with a UL adaptor (Brookfield LVDVE, MA, USA). The viscosity-average molecular weight of the PAM was determined to be 17.55 MDa using the Mark-Houwink equation and dilute-solution viscosity principles, demonstrated in the Supplementary Information. The range of shear rates studied corresponds to the maximum possible range given the limitations of the viscometer, namely less than 10% torque is considered inaccurate, limiting the lower end of the shear rate range, and the maximum speed of the viscometer is 100 RPM, limiting the upper end of the range.

### 2.2 Bacterial culture

Magnetospirillum magneticum strain AMB-1 (ATCC 700264) was used for magnetotaxis experiments. Revised magnetic Spirillum growth medium (ATCC medium 1653) was used to grow a liquid culture of M. magneticum. The medium consists of 10.0 mL Wolfe’s vitamin solution, 5.0 mL of Wolfe’s mineral solution, 2.0 mL ferric quinate, 0.45 mL resazurin, 0.68 g KH2PO4, 0.12 g NaNO3, 0.035 g ascorbic acid, 0.37 g tartaric acid, 0.37 g succinic acid and 0.05 g sodium acetate in 1.0 L distilled water. NaOH was used to adjust the pH of the medium to 6.75. Prior to experiments, the growth medium was inoculated in a 50% ratio with M. magneticum in a volume of 20 mL, and the cells were incubated at 30°C for 48 h. The vials were closed with minimal headspace to create microaerobic conditions. Resazurin was included as a reduction–oxidation potential indicator, which causes the solution to turn from pink to clear as the cells consume oxygen. Experiments were conducted after the solution turned clear since this change indicates cell growth.

### 2.3 Microfluidic device fabrication

The microfluidic devices were comprised of a single channel 50 μm deep and 300 μm wide. Microfluidic devices were fabricated using polydimethylsiloxane (PDMS, Dow Silicones Corporation, Midland, MI, USA) using standard soft lithography and replica molding techniques.(McDonald et al., 2000) A master was fabricated with photolithography (Neutronix-Quintel NxQ4006) on a single-side-polished silicon wafer with SU-8 photoresist (Kayaku, Westborough, MA, USA). The PDMS was mixed in a 10:1 w/w ratio of base and curing agent then degassed for 30 min. The PDMS was poured into the mold, degassed, and then cured at 75°C for 30 min. The cured PDMS was demoulded, then a biopsy puncher was used to create holes for the inlets and outlets. The surfaces of the PDMS and a glass microscope slide were oxidized via plasma oxidation in a plasma cleaner (Harrick Plasma, Ithaca, NY, USA). The two surfaces were immediately placed in contact to form a Si-O-Si bond.

### 2.4 Microscopy

An inverted epi-fluorescent microscope (Olympus IX83, Tokyo, Japan) with a low dark noise high-speed CMOS camera (Zyla-4.2-CL10, ANDOR, Belfast, Ireland) was used to record brightfield videos of MTB magnetotaxis in the device. Videos were recorded at room temperature using 40× magnification (NA 0.6 LUCPlanFL N, Olympus, Tokyo, Japan) at 10 FPS. Images and videos were processed using ImageJ 1.54f, and the ImageJ Manual Cell Tracking plugin was used to track the individual cells. Only motile cells were considered in the analysis. Data analysis, data visualization and statistical analysis were performed with a custom Python code.

### 2.5 Magnetotactic navigation of MTB

**Figure 1:**
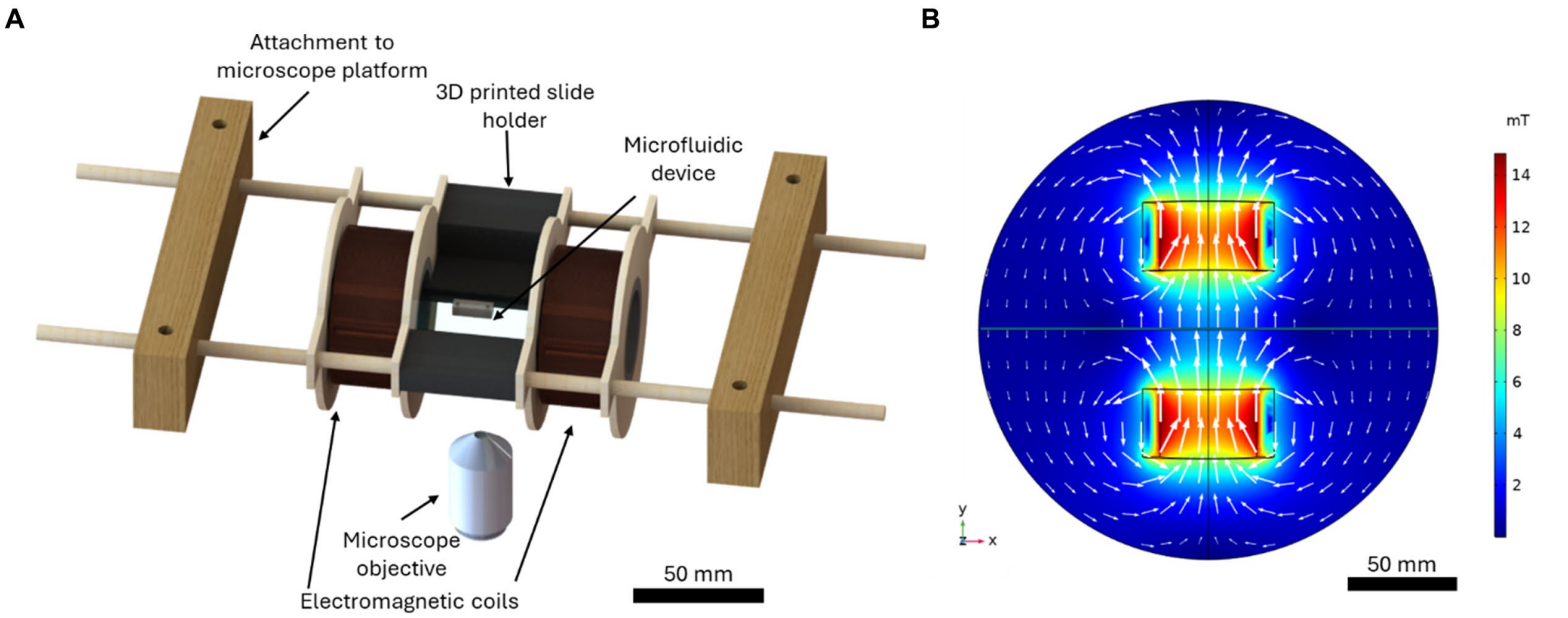
**(A)** The computer-aided design of the microfluidic-electromagnetic coils platform used to produce the magnetic field for the magnetotaxis studies. **(B)** The simulated magnetic flux density produced by the coils for a current of 1.3 A, the current used in experiments. The white arrows indicate the direction and magnitude of the magnetic flux.

A custom microfluidic-electromagnetic coils platform (Bradley et al., 2023) was used to produce a linear 4.0 mT magnetic field for MTB magnetotaxis studies. The computer-aided design (CAD) produced in SolidWorks 2022 (Dassault Systèmes, France) is shown in Figure 1A. The platform, with a total dimension of 236 mm, consisted of two coils with a radius of 21 mm, width of 30 mm and a stacked wire thickness of 7.7 mm separated by 51.3 mm and a central area for placing microfluidic chips with complete open access and spatial clearance between coils. The electromagnetic coils were designed using computer-aided design (CAD) tools. The centres of the coil spindles were made with 1.5-inch diameter PVC pipe, and the sides were made from 1/8-inch thick plywood. The plywood was cut such that there were two support structures on either side to be used to suspend the coils. Copper magnetic wire (22 AWG, Digi-Key Electronics, Thief River Falls, MN, USA) was used with 400 turns on each coil. A microscope slide holder that fits standard microscope slides of 25 mm × 75 mm, which also serves as a spacer for the coils, was 3D printed using a stereolithography (SLA) technique (Photon S, ANYCUBIC, Shenzhen, China). Wooden blocks were attached to the microscope platform using screws, and wooden dowels were inserted between the blocks. The coils and microscope slide holder were attached to the wooden dowels such that they were suspended above the microscope objective. The coils could slide along the dowels to adjust the separation if needed. The separation of the coils for this characterization was 81 mm, centre-to-centre, corresponding to the smallest separation distance possible to accommodate the microscope components. The wires were connected to a 30 V 10 A DC power supply (Sky TopPower, STP3010H, Shenzhen, China). The magnetic field produced by the coils was measured experimentally using a Hall effect gaussmeter (F.W. Bell 5,070, F.W. Bell, Portland, Oregon). The magnetic field produced by the coils was predicted using COMSOL Multiphysics 5.6 software (COMSOL, Inc., Sweden). Figure 1B presents the magnetic flux density produced by the coils for the current used experimentally, 1.3 A.

### 2.6 Experimental procedure

A stock solution of 10 mg/mL PAM dissolved in the MTB growth medium was prepared 12 h prior to experiments. The PAM stock solution was diluted to the desired concentration by mixing the MTB in the liquid growth medium. The PAM-MTB solution was immediately injected into the microfluidic device after mixing. The inlets and outlets of the device were sealed to stop the fluid flow, then the device was immediately loaded into the electromagnetic-microfluidic platform, attached to the microscope. The time between when the cells were mixed with PAM to when the cells were studied was 3 min. The magnetic field was turned on to 4 mT, and the videos of the bacteria swimming in the channel were recorded for 5 min at 10 fps, at a focal point 15 μm above the bottom of the channel. Videos with flows greater than 10% of the average MTB swimming speed were not included in the study. The total experimental time was 8 min including 5 min for video acquisition. Experiments were repeated 2–8 times depending on the number of motile cells in the sample, to achieve a sample size of 60 cells per condition (240 cells studied total).

### 2.7 Statistical analysis

One-way ANOVA tests were used to determine if there was a significant difference in the swimming speed and the influence of cell size on their motility between the bacterial groups swimming in PAM with different concentrations. A Tukey’s Honestly Significant Difference (HSD) test, which provides the critical value that determines the minimum difference between group means to be considered statistically significant, was used to test the statistical difference in swimming speeds and motility based on cell size, between bacterial groups. The coefficient of determination, *R*^2^, was used to assess the influence of the PAM concentration on the linearity of the bacterial magnetotactic motility. Although relatively high values of linearity are expected under directed (magnetotactic) motion, a decrease in *R*^2^ value indicates a decrease in the linearity of the trajectory. The whiskers in the box plots correspond to the standard interquartile range from the lower and upper quartile.

## 3 Results and discussion

**Figure 2:**
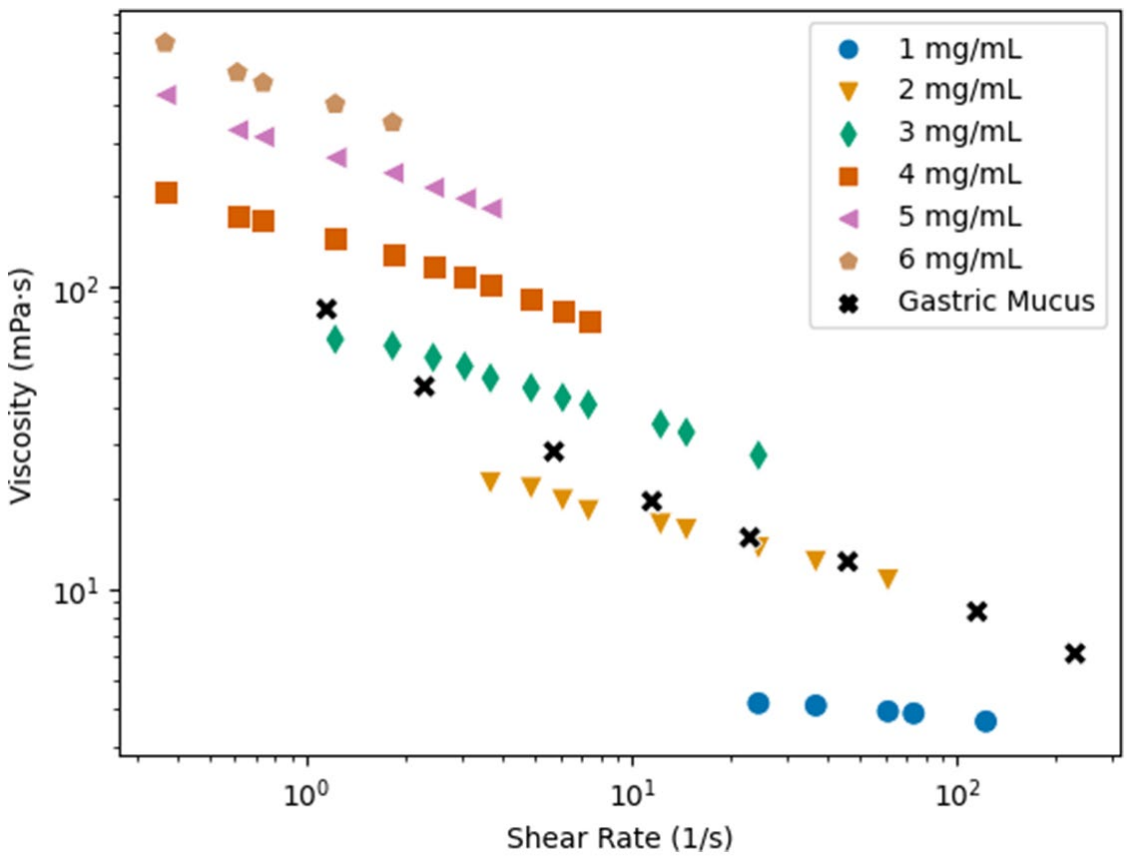
Shear rate vs. viscosity of PAM in concentrations of 1–6 mg/mL and gastric mucus (Curt and Pringle, 1969).

Polymer solutions were used in the microfluidic channels to mimic the non-Newtonian biological fluids. Poly(acrylamide) (PAM) was used because this polymer solution has similar rheological properties to mucus and it has been used previously to study sperm cells in non-Newtonian medium (Tung et al., 2017; Hyakutake et al., 2018). The viscosity of the PAM solutions was characterized for concentrations of 1–6 mg/mL to determine the concentration that is most similar to biological mucus. Figure 2 presents the viscosity vs. shear rate results, plotted with data for the viscosity vs. shear rate of gastric mucus (Curt and Pringle, 1969). All concentrations of the PAM solution exhibited shear-thinning flow behavior, the viscosity decreased as the shear rate increased. It is important to note that for the results of this study, only the shear thinning effects are considered, but other rheological properties may influence the swimming kinematics of the microswimmers (Patteson et al., 2015). The 2 mg/mL PAM solution had the most similar rheological behavior to the gastric mucus when comparing the viscosity vs. shear rate data, and the 3 mg/mL solution was similar at lower shear rates. Typical shear rates of swimming bacteria are on the order of 10^0^ s^−1^ (Figueroa-Morales et al., 2019). Since these two concentrations are the closest representation of biological mucus, they will be the concentrations of interest for this study.

**Figure 3:**
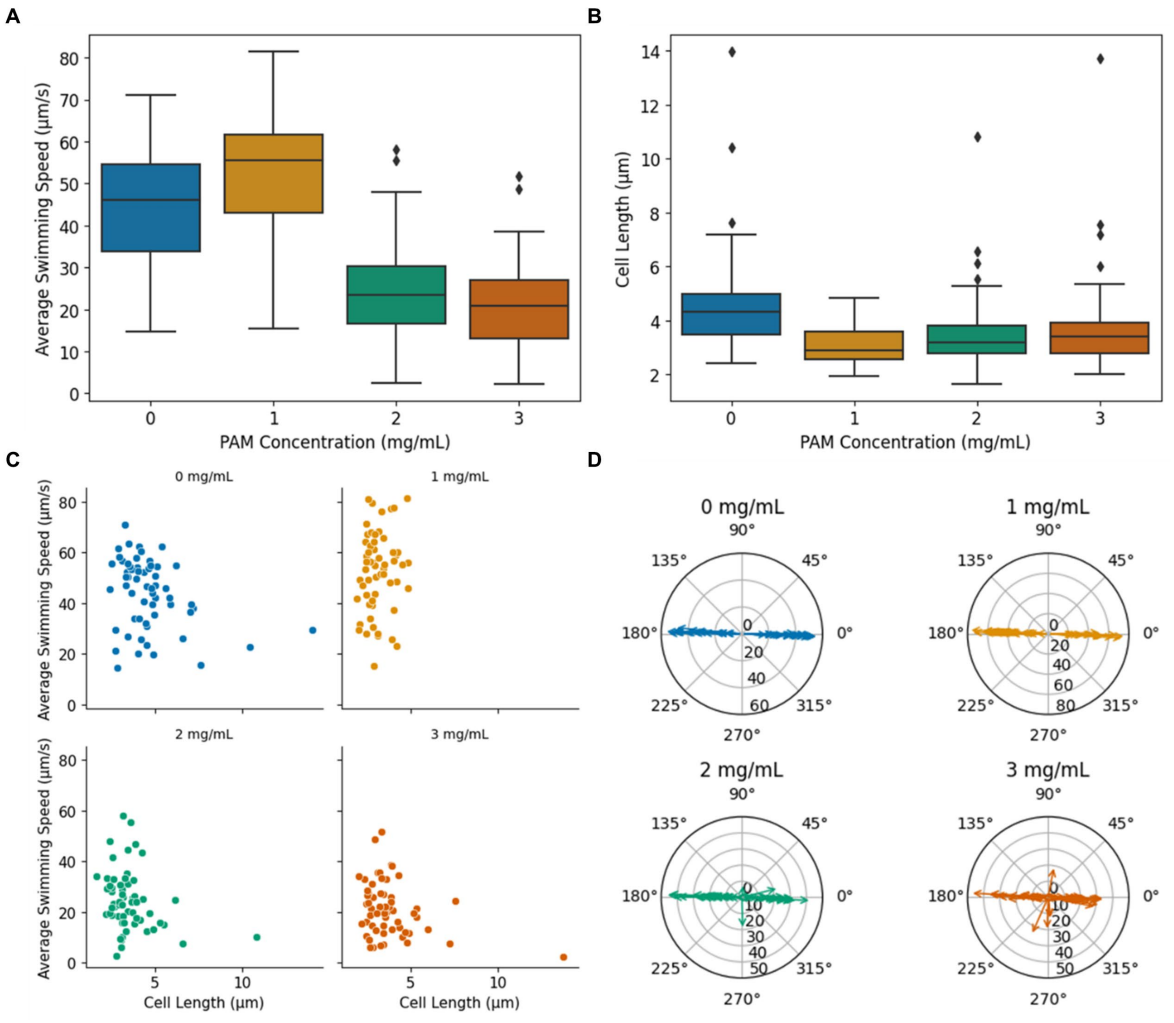
**(A)** Average bacteria swimming speed vs. PAM concentration. **(B)** Cell length vs. PAM concentration. The diamond markers indicate outliers. **(C)** The average swimming speed vs. cell length. **(D)** Radial plots indicating the overall trajectory angle within the ROI for each PAM concentration. The magnitude of the arrows indicates the average swimming speed during the trajectory in μm/s.

A key indicator of bacterial motility in non-Newtonian fluids is swimming speed. Figure 3A presents the average speed of MTB in PAM solutions with concentrations ranging from 0 to 3 mg/mL (*n* = 60 for each condition), where 0 mg/mL corresponds to their usual growth medium, a Newtonian fluid. The average swimming speed for each cell was calculated from the average of the frame-to-frame speeds for each cell’s path within the region of interest (ROI). When swimming in the growth medium, MTB exhibited a mean swimming speed of 44.0 ± 13.6 μm/s. This speed is consistent with literature findings for the magnetospirillum genus swimming speed in growth medium under stagnant conditions (Seong and Park, 2001; Popp et al., 2014; Rismani Yazdi et al., 2018). The mean swimming speed increases significantly from 44.0 ± 13.6 μm/s in 0 mg/mL of PAM to 52.73 ± 15.6 μm/s in 1 mg/mL of PAM (*p* = 0.0017), then decreases significantly to 24.51 ± 11.7 μm/s in 2 mg/mL of PAM (*p* < <0.01). The mean swimming speed in 3 mg/mL of 21.23 ± 10.5 μm/s is not significantly difference between the mean swimming speed in 2 mg/mL. A one-way ANOVA revealed that there was a statistically significant difference in mean swimming speed between at least two PAM concentrations (*p* < <0.01). Using Tukey’s HSD test for multiple comparisons, we found that the mean swimming speed was significantly different between all groups except the mean swimming speed between 2 and 3 mg/mL are not significantly different (*p* = 0.511). The observed trend of an initial speed increase with viscosity increase followed by a decrease in speed after a characteristic viscosity value is consistent with previous reports for many types of motile, flagellated bacteria in polymeric fluids (Schneider and Doetsch, 1974; Berg and Turner, 1979; Martinez et al., 2014). While the mechanisms behind this behavior remain unclear, it has been proposed that this initial increase in swimming speed is related to the nature of the polymer chains’ structure (Lauga, 2016; Zöttl and Yeomans, 2019). The long, unbranched chain-polymers are expected to form a network against which the cells can push and create a non-uniform distribution of polymers in the vicinity of the bacterium body and flagella, leading to an apparent slip (Lauga, 2016; Zöttl and Yeomans, 2019).

The cell length of the motile bacteria was recorded to investigate the influence of cell size on MTB motility in non-Newtonian fluids, and the summary of the data is presented as a boxplot in Figure 3B. Since only motile bacteria were considered in this study, the measurements correspond to cells that were capable of swimming in the given condition. In 0 mg/mL of PAM, the mean cell length was 4.7 ± 1.9 μm. The mean cell length decreased in 1 mg/mL of PAM to 3.1 ± 0.7 μm. The mean cell length for 2 and 3 mg/mL of PAM was 3.5 ± 1.3 μm and 3.8 ± 1.7 μm, respectively. A one-way ANOVA test indicated an overall significant difference between groups (*p* < <0.01). Using the Tukey’s HSD test to compare the means between the groups, the mean cell length was significantly different when comparing the 0 mg/mL group to all other groups (*p* < <0.01, *p* = 0.0002, 0.0059 for 1, 2, and 3 mg/mL, respectively). Contrarily, the mean cell length was not significantly different between 1 and 2, 1 and 3, and 2 and 3 (*p* = 0.51, 0.096, 0.79, respectively). That is, the mean cell length in the Newtonian, growth medium decreased in the PAM solution, but did not significantly change as the concentration increased, demonstrating that, on average, longer cells were capable of swimming in the Newtonian fluid compared to the non-Newtonian polymer solution. In 0, 2, and 3 mg/mL PAM solutions, larger cells, outliers represented by the diamond markers in Figure 3B, were observed with a maximum cell length of 14.0, 10.8, and 13.7 μm, respectively. These larger cells were not observed in the 1 mg/mL group, which had a maximum cell length of 4.9 μm. The cell length and the average swimming speed of each bacterium was plotted in Figure 3C. A linear regression analysis was used to test if the length of the cell explained the average speed. The results of the regression showed a very weak correlation between cell length and swimming speed in all concentrations (*R*^2^ = 0.10, 0.018, 0.071, 0.092 for 0, 1, 2, and 3 mg/mL PAM, respectively). The potential conclusions that may arise from such low *R*^2^ values require caution as they indicate an extremely, almost null correlation between the independent and dependent variables, suggesting that the model cannot be used to predict linearity. In the context of this study, the very small *R*^2^ values suggest that the length of the cells had little to no influence on the swimming speed in any of the fluids tested in this study.

The ability to effectively direct the MTB with an applied magnetic field is imperative for their use in biomedical applications. Since the applied magnetic field from the electromagnetic coil platform produces linear field lines, parallel to the length of the microfluidic channel and horizontally along the ROI, efficient magnetotaxis would imply a linear, horizontal trajectory. Radial plots of the bacteria’s overall trajectory angle, calculated from the start and end positions for each cell within the ROI, are presented in Figure 3D. The magnitude of the arrows represents the swimming speed of the individual bacterium. With magnetic field lines aligned horizontally in the ROI, it is expected that the bacteria will navigate with an angle of 0° or 180°, since *M. magneticum* AMB-1 exhibit axial magnetotaxis, navigating in both directions along the field lines (Bazylinski et al., 2013). In both 0 and 1 mg/mL of PAM, all tracked bacteria followed the magnetic field lines in both directions with a maximum deviation from the horizontal of 5.3° and 3.3°, respectively. In 2 and 3 mg/mL of PAM, the percentage of cells with a trajectory angle greater than 5° away from the horizontal increased with PAM concentration, as 5 and 20% of cells, respectively. This increase in cells that have an overall trajectory that does not follow the magnetic field lines indicates that the polymer solution could interfere with the ability to direct their navigation via magnetotaxis.

**Figure 4:**
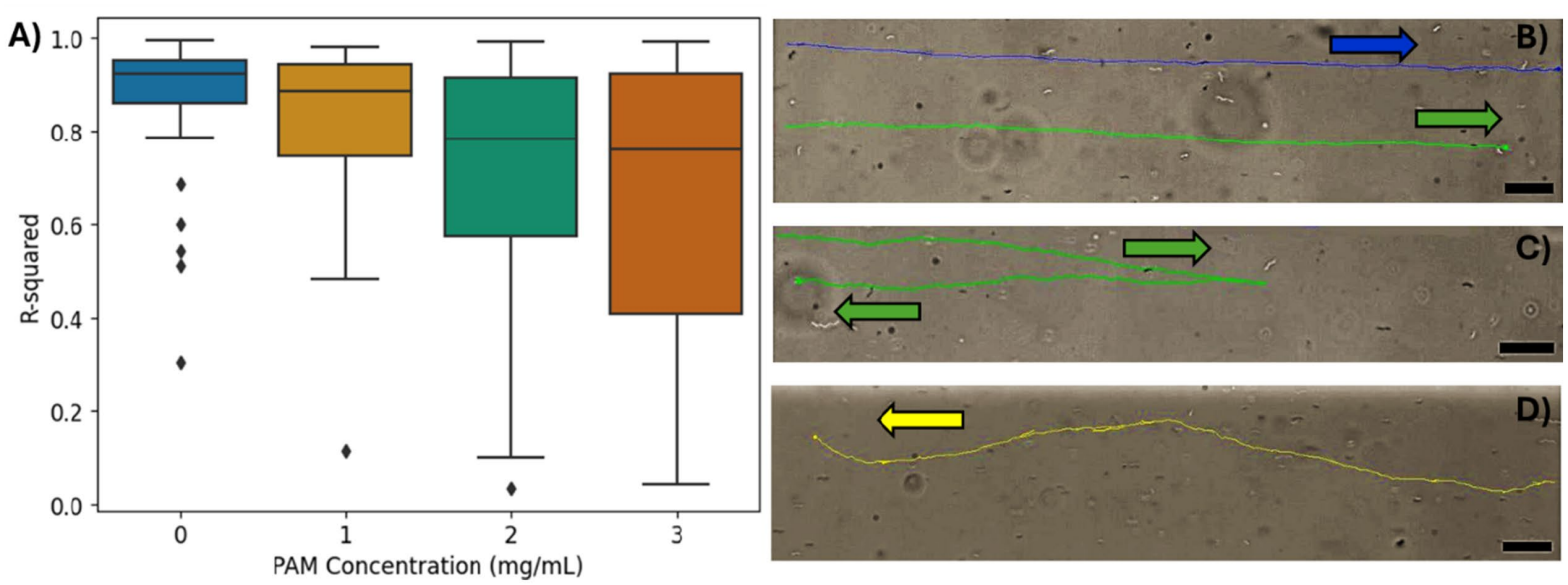
**(A)** The *R*^2^ values from the linear regression of the bacteria trajectories for each PAM concentration. **(B–D)** The tracking overlays of individual bacteria. Arrows indicate swimming direction. Scale bar = 20 μm. **(B)** An example of high *R*^2^, linear trajectories in 0 mg/mL PAM solution. The top trajectory in blue has an *R*^2^ = 0.92 and the bottom trajectory in green has an *R*^2^ = 0.94. **(C)** An example of a single reversal resulting in an *R*^2^ value of 0.10 in 3 mg/mL PAM solution. **(D)** An example of a ‘wavy’ trajectory with a several short reversals that do not impact the overall direction of motion. *R*^2^ = 0.34 in 2 mg/mL PAM solution.

To further investigate the magnetotaxis effectiveness, a linear regression was performed on the coordinates of each cell’s path within the ROI to quantify the linearity of the MTB trajectory. The coefficient of determination (*R*^2^) was used to approximate the linearity of the coordinate points for the trajectory of each cell, and the distribution of *R*^2^ values for the bacteria in each PAM concentration are summarized in Figure 4A. A limitation of an *R*^2^ analysis is that it cannot be applied to a horizontal dataset since, in this case, the y values do not have a dependency on the x values. Due to an imperfect alignment of the microfluidic channel, magnetic field lines, and microscope camera, most trajectories are not exactly horizontal, so the *R*^2^ analysis was possible. However, some datasets were too close to horizontal for an effective *R*^2^ analysis, so trajectories with a slope less than 0.01 were excluded from this analysis. The mean *R*^2^ values for 0, 1, 2, and 3 mg/mL of PAM, respectively, were 0.88 ± 0.13, 0.83 ± 0.17, 0.72 ± 0.26, and 0.68 ± 0.29. As the PAM concentration increased, the mean *R*^2^, indicating the linearity of the trajectories decreased and the standard deviation increased. Figure 4B demonstrates linear trajectories with *R*^2^ values of 0.92 (blue) and 0.94 (green). Possible trajectory patterns that would result in less linearity, include a ‘curved’ or ‘wavy’ path, and reversal of swimming direction. Figure 4C shows a cell that appears to be following the magnetic field lines but reverses its’ direction resulting in an *R*^2^ value of 0.10. Figure 4D shows an example of a cell with both a ‘wavy’ trajectory and 14 reversals, resulting in an *R*^2^ value of 0.34.

**Figure 5:**
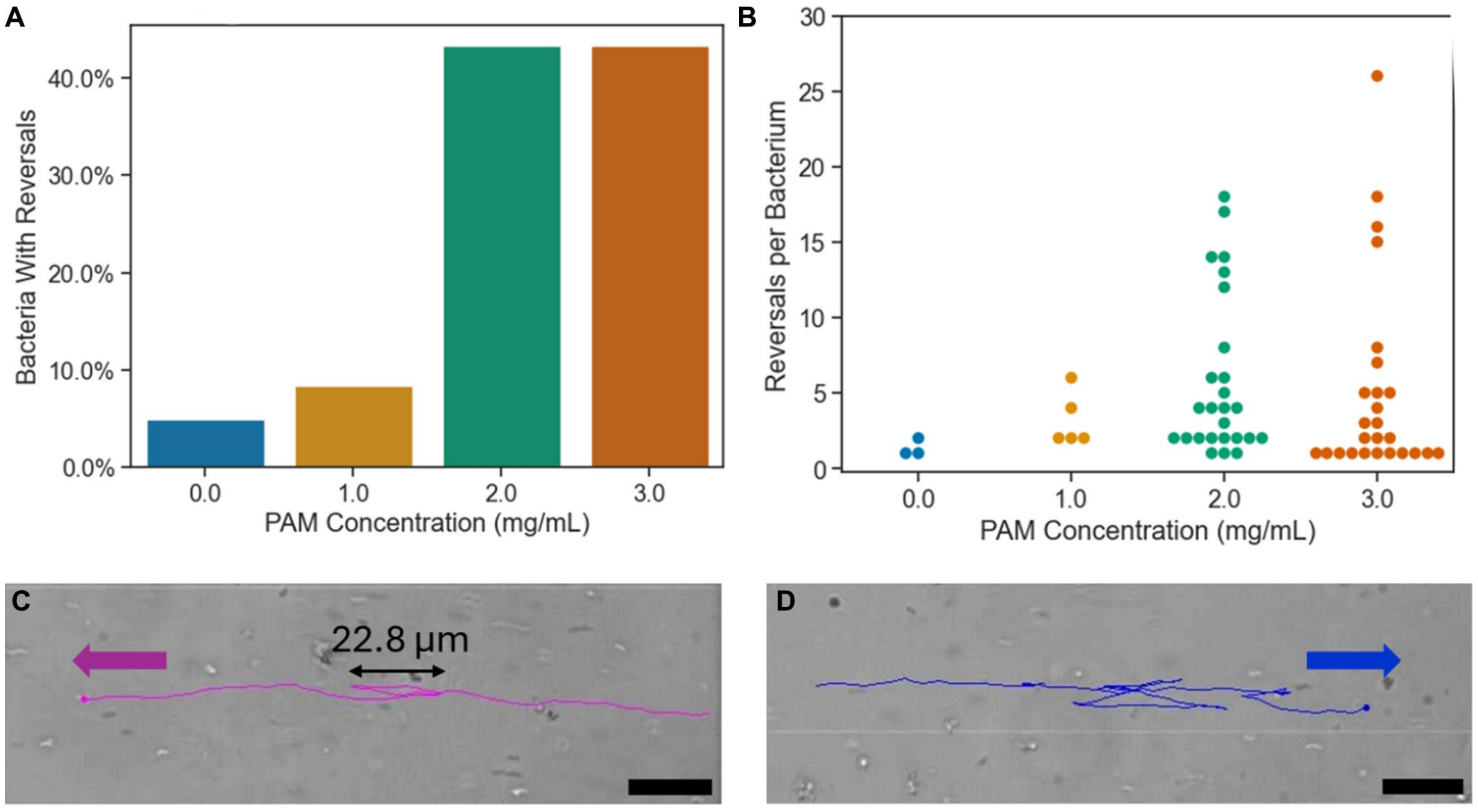
**(A)** The percentage of bacteria with at least one reversal of direction during their trajectory for each PAM concentration. **(B)** The number of reversals per bacterium for each PAM concentration. Each point represents a single bacterium. **(C)** The tracking overlay of a bacterium in 2 mg/mL PAM solution with two reversals in its’ trajectory. **(D)** The tracking overlay of a bacterium in 2 mg/mL PAM solution with 14 reversals in its’ trajectory. Scale bar = 20 μm.

Run and reverse motion has been observed in *magnetospirillum* genus, where the bacterium reverses its’ direction of motion, without rotating its’ body, as opposed to the run and tumble motion, seen commonly in *E. coli*, where the cell body rotates an average of 68° during the “tumble” motion (Berg and Brown, 1972; Popp et al., 2014). With axial magnetotaxis exhibited by the *magnetospirillum* genus, the magnetic field lines act as an axis of orientation that the bacteria navigate along in both directions with frequent, spontaneous reversals of swimming direction along this axis (Lefèvre et al., 2014). In Figure 5A, the percentage of bacteria that reversed their swimming direction at least once during their trajectory in the ROI is shown for each PAM concentration. The bars represent a whole number of cells that correspond to a bacteria population percentage. In the growth medium, corresponding to 0 mg/mL of PAM, 5.0% of bacteria had a reversal in their trajectory. In 1 mg/mL of PAM, this increased slightly to 8.3%, then in 2 and 3 mg/mL of PAM, the percentage of bacteria with a reversal increased to 43.3% for both samples. The increase from less than 10% of the sample reversing their swimming direction to 43.3% with a PAM concentration above 2 mg/mL corresponds with the threshold polymer concentration where the swimming speed decreases shown in Figure 3. Figure 5B presents the number of times a bacterium reverses its’ direction within the ROI. The maximum number of reversals for 0 and 1 mg/mL of PAM was 2 and 6, respectively. This maximum increases to 18 for 2 mg/mL and 26 for 3 mg/mL. Not only does the number of bacteria exhibiting reversals increase with a PAM concentration above 1 mg/mL, but so does the number of reversals per bacterium, resulting in a decreased efficiency in directed motion toward the target location. An example of a bacterium with 2 reversals is shown in Figure 5C and a bacterium with 14 reversals is shown in Figure 5D.

The explicit cause(s) of a bacterium reversing its’ direction is not well understood. However, studies have shown the morphology of the bacteria, specifically the flagella number and placement, enables it to reverse its’ direction (Zhang and Wu, 2020; Grognot and Taute, 2021). *M. magneticum* AMB-1 has amphitrichous flagella, a single flagellum on each pole of the cell body (Zhang and Wu, 2020). The bipolar flagella rotate in opposite directions to propel the cell forward, then reverse their orientation for the cell to move in the opposite direction (Murat et al., 2015). The run-reverse swimming pattern has been observed in other amphitrichous bacteria, including *Campylobacter jejuni* (Grognot and Taute, 2021). It has been shown that swimming reversals can be a result of a physical stimulus in their environment such as a local oxygen concentration, the oxygen gradient, or variation in the magnetic field (González et al., 2014; Lefèvre et al., 2014). Additionally, the MTB have been shown to repeatedly reverse their swimming direction when interacting with flat surfaces (Rismani Yazdi et al., 2018). In this study, the increased observation and frequency of reversals in the MTB trajectories in higher concentrations of PAM suggests that the cells are influenced by the increased viscosity or potential heterogeneities in the solution. Further studies in other non-Newtonian solutions, such as with different polymer chain lengths and elasticities, would be beneficial to improve the understanding of the cause of the swimming reversals in the non-Newtonian polymer solutions.

## 4 Conclusion

An understanding of MTB motility and directed navigation through non-Newtonian fluids is needed for their effective use in targeted drug delivery applications where they may encounter biological non-Newtonian fluids such as blood plasma or mucus. In this work, we investigated the influence of 3 concentrations of polyacrylamide, a mucus-mimicking non-Newtonian polymer solution. The influence of cell length on the average swimming speed of each bacterium was compared. A weak correlation between cell length and swimming speed in all concentrations was found. The swimming speeds increase significantly when the PAM concentrations are increased, from 44.0 ± 13.6 μm/s in 0 mg/mL to 52.73 ± 15.6 μm/s in 1 mg/mL but decrease significantly to 24.51 ± 11.7 μm/s in 2 mg/mL. A non-significant speed decrease to 21.23 ± 10.5 μm/s was found in a PAM concentration of 3 mg/mL. This trend of a speed increase in low polymer concentrations followed by a decrease in speed as the concentration increases past a threshold concentration is analogous to other studies of motile, flagellated bacteria (Schneider and Doetsch, 1974; Berg and Turner, 1979; Martinez et al., 2014). Past this threshold concentration of PAM, there is a higher percentage of cells with an overall trajectory angle deviating from the angle of the magnetic field lines of 5 and 20% of cell in 2 mg/mL and 3 mg/mL, respectively. There is also less linearity in the trajectories with increasing concentration of PAM, evidenced by the decreasing mean *R*^2^ values for the groups, indicating the linearity of the trajectories. There was also an increase in reversals of swimming direction both in the number of bacteria exhibiting reversals and the number of reversals per bacterium, resulting in a decreased efficiency in directed motion toward the target location. To compliment the work in this article, other rheological properties should be studied, such as fluid elasticity and the polymer chain length, to have a more complete understanding of additional factors that influence the swimming behaviors of MTB in non-Newtonian fluids. Altogether, this work demonstrates that MTB can be directed using an applied magnetic field in polymer concentrations mimicking biological mucus and demonstrates that the viscosity of the medium in which the bacteria are transported influences the linearity of their trajectories which alters the effective path that could be predefined in Newtonian fluids when transport if achieved by magnetotaxis.

## Data availability statement

The raw data supporting the conclusions of this article will be made available by the authors, without undue reservation.

## Author contributions

BB: Conceptualization, Data curation, Formal analysis, Investigation, Methodology, Validation, Visualization, Writing – original draft, Writing – review & editing, Resources. CE: Funding acquisition, Project administration, Resources, Supervision, Writing – review & editing, Conceptualization, Formal analysis, Investigation, Methodology.
