# Supplementary material for: Single-cell magnetotaxis in mucus-mimicking polymeric solutions
Source: Front Microbiol. 2024 Jul 18;15:1436773. doi: 10.3389/fmicb.2024.1436773 (PMC11293504; doi:10.3389/fmicb.2024.1436773)
Supplement: Supplementary file 1 [file Data_Sheet_1.PDF]

Supplementary Information: PAM Molecular Weight Characterization

The viscosity average molecular weight of the polymer was determined using dilute solution viscosity principles. The viscosity was measured for shear rates from 7.34-122.3 s^{-1} for concentrations from 0.5-2.5 mg/mL. The reduced viscosity, η_{red}, was calculated using Equation 1 (Anandha Rao, 2014).

$$\eta_{red} = \frac{\eta - \eta_0}{c \cdot \eta_0} \quad (1)$$

where η is the absolute viscosity of the solution, η_0 is the viscosity of the solvent, and c is the concentration of polymer in solution.

The intrinsic viscosity, $[\eta]$, was calculated using Equation 2 (Anandha Rao, 2014).

$$[\eta] = \lim_{c \to 0} \eta_{red} = \lim_{c \to 0} \frac{\eta - \eta_0}{c \cdot \eta_0} \quad (2)$$

such that the y-intercept of the reduced viscosity vs concentration plot is the intrinsic viscosity. The reduced viscosity was plotted with respect to concentration for each shear rate, and the average y-intercept of all the shear rates was found (**Figure SI-1**).

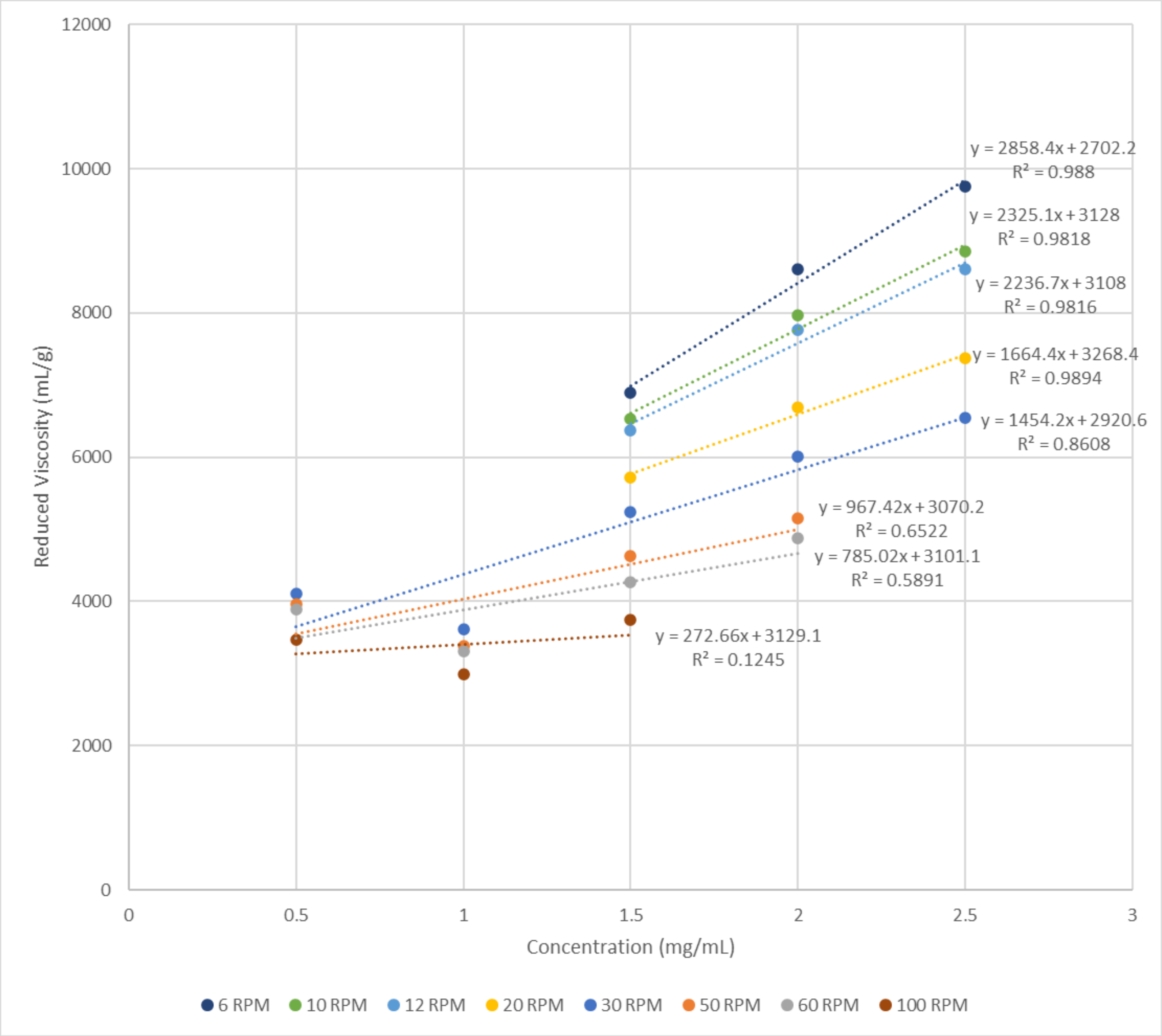

Figure SI-1. *A plot of the reduced viscosity vs concentration for FLOPAM VHM.*

The viscosity average molecular weight, M_V, was then calculated using the Mark-Houwink equation (Equation 3). (Anandha Rao, 2014)

$$[\eta] = kM_V{}^a \tag{3}$$

where k and a are the Mark-Houwink constants which depend on the solvent-polymer pair. For polyacrylamide and water, $k = 3.09 \cdot 10^4$ and $a = 0.67$ at 20°C (Orwoll & Chong, 1999). The intercept of each trendline corresponds to the reduced viscosity, which was used in Equation 3 to calculate the viscosity average molecular weight. The average of these values was found to be 17.55 MDa for FLOPAM VHM.

References

Anandha Rao, M. (2014). Introduction: Food Rheology and Structure. In *Rheology of Fluid, Semisolid, and Solid Foods* (3rd ed., pp. 1–27). Springer.

Orwoll, R. A., & Chong, Y. S. (1999). Polyacrylamide. In J. E. Mark (Ed.), *Polymer Data Handbook* (1st ed., pp. 247–251). Oxford University Press.
